# Skin as a Metabolic Organ: Dermatologic Markers of Morbid Obesity and Their Role in Risk Stratification and Treatment Monitoring

**DOI:** 10.3390/diagnostics16091314

**Published:** 2026-04-27

**Authors:** Aleksandra Sado, Monika Tomaszewska, Simona Wójcik, Anna Rulkiewicz

**Affiliations:** 1 Miedzyleski Specialist Hospital, 04-749 Warsaw, Poland; 2 Wyższa Szkoła Nauk Medycznych, 02-678 Warsaw, Poland; 3 LUX MED, 02-676 Warsaw, Poland

**Keywords:** morbid obesity, skin manifestations, metabolic disorders, risk assessment, acanthosis nigricans

## Abstract

Morbid obesity is a chronic condition characterized by metabolic disorders and low-grade chronic inflammation, both of which are closely linked to insulin resistance and adipokine dysregulation. In addition to its systemic effects, obesity also leads to structural and functional changes in the skin, supporting its role as an active metabolic and immunological organ. This study analyzed skin lesions occurring in individuals with morbid obesity and explored their potential relevance in the context of metabolic risk and treatment response rather than establishing clinically validated tools. The focus was on how excess adipose tissue affects the skin through metabolic, hormonal and mechanical mechanisms. Although this review focuses on morbid obesity, many of the included studies examine general obesity without separating its severity. Therefore, the findings may not fully reflect patients with BMI ≥ 40 kg/m^2^ and should be interpreted with caution. Three main areas were considered: the pathophysiological mechanisms underlying obesity-related skin lesions, selected dermatological manifestations as potential markers associated with metabolic risk, and changes in these manifestations during pharmacological, surgical, and lifestyle interventions. Available studies show that acanthosis nigricans and multiple acrochordons are consistently associated with insulin resistance, metabolic syndrome, and metabolic dysfunction-associated steatotic liver disease. An increase in BMI is also associated with impairment of the epidermal barrier, changes in the composition of skin lipids, and modifications of the skin microbiome, while biomechanical factors promote the development of chronic inflammation in the intertriginous areas. It has been shown that normalization of metabolic parameters achieved through GLP-1-based pharmacotherapy, bariatric surgery, or lifestyle changes can improve some skin manifestations, especially acanthosis nigricans. However, it should be emphasized that most available data are based on cross-sectional or observational studies, and validated composite dermatological indices are still unavailable. Skin changes in patients with morbid obesity often reflect underlying metabolic and hormonal disturbances. They may have potential as additional, non-invasive clinical clues, but they should not be treated as independent tools for risk assessment or treatment monitoring. At present, most evidence shows associations only, and it is unclear whether these findings add meaningful predictive value beyond standard metabolic markers. More prospective studies are needed to confirm their clinical usefulness and to define their role in assessing metabolic risk and monitoring treatment over time.

## 1. Introduction

### 1.1. Morbid Obesity: Clinical and Metabolic Background

According to the World Health Organization, overweight and obesity are defined as abnormal or excessive fat accumulation that presents a risk to health. In clinical practice, body mass index (BMI) is used for classification, with values ≥25 kg/m^2^ indicating overweight and ≥30 kg/m^2^ indicating obesity [1]. Morbid obesity (class III obesity) is defined as BMI ≥ 40 kg/m^2^ regardless of comorbidities, or BMI ≥ 35 kg/m^2^ in the presence of obesity-related metabolic, cardiovascular, or mechanical complications [2].

Obesity is currently recognized as a multifactorial, chronic, relapsing, non-communicable disease characterized by dysfunctional and/or excessive accumulation of body fat with direct adverse effects on organ function and systemic health. Contemporary clinical frameworks emphasize that management should not be limited to weight reduction alone but should adopt a long-term, complication-oriented approach integrating behavioral interventions, pharmacotherapy, and metabolic or bariatric procedures [3]. In alignment with this model, treatment strategies are increasingly guided by the presence and severity of obesity-related complications rather than BMI alone [4].

Morbid obesity is characterized by adipose tissue dysfunction and chronic low-grade inflammation. Increased production of pro-inflammatory cytokines, including tumor necrosis factor-α (TNF-α) and interleukin-6 (IL-6), interferes with insulin signaling pathways and contributes to systemic insulin resistance [5]. This immunometabolic disturbance is associated with an increased risk of cardiovascular disease, type 2 diabetes mellitus, metabolic dysfunction-associated steatotic liver disease, and renal complications such as albuminuria and chronic kidney disease [6].

Current diabetes care standards provide structured diagnostic and classification criteria based on glycemic thresholds, including fasting plasma glucose, oral glucose tolerance testing, and glycated hemoglobin (HbA1c), forming the basis for risk stratification and management algorithms. These structured frameworks rely predominantly on biochemical and clinical parameters. Cutaneous manifestations are not included in formal diagnostic or risk stratification criteria within these standards [7].

The aim of this review is not to replace standard biochemical risk assessment but to determine whether selected skin findings may serve as simple clinical signals prompting further metabolic evaluation and to explore which of these changes may improve alongside insulin resistance and systemic inflammation during treatment.

Beyond its role in energy storage, adipose tissue exerts endocrine, inflammatory and immune-modulatory effects that influence multiple organ systems [3]. These systemic alterations provide the biological context for examining peripheral tissues, including the skin, as potential sites reflecting underlying metabolic dysregulation.

For clarity, this review distinguishes between mechanistic pathways linking obesity to skin alterations and clinically observable dermatologic manifestations. These are discussed separately, and only findings supported by human studies are considered in the context of potential clinical relevance.

### 1.2. Skin as a Metabolic Organ

The skin is increasingly recognized as a neuroendocrine organ capable of synthesizing and metabolizing steroid hormones, neuropeptides, and other signaling molecules, thereby participating in systemic endocrine regulation [8]. Adipocytes located in the dermis and subcutaneous tissue participate in lipogenesis and lipolysis and produce adipokines such as leptin, adiponectin, and resistin, which regulate local inflammatory and regenerative processes within the skin [9,10]. Dermal white adipose tissue has been shown to contribute to thermogenic responses and cold-induced adaptation, representing an additional component of cutaneous metabolic function [11].

In addition to its metabolic properties, the skin performs important immunological functions. Keratinocytes and resident antigen-presenting cells cooperate to maintain inflammatory balance and respond to environmental stimuli [12]. An integral component of the cutaneous system is the skin microbiota, which interacts with the host immune system and contributes to epidermal barrier function and antimicrobial defense [13].

This integrative perspective provides a biological rationale for exploring dermatologic manifestations of morbid obesity as potential indicators of systemic metabolic burden. Although this review focuses on morbid obesity, it should be acknowledged that a substantial proportion of the available evidence derives from studies that include broader obesity populations without stratification by severity.

## 2. Materials and Methods

This manuscript is a narrative review that summarizes current evidence on skin changes associated with morbid obesity and their possible role in assessing metabolic risk and monitoring treatment.

A targeted literature search was performed in PubMed/MEDLINE and Scopus, with additional support from Google Scholar. The search covered studies published from January 2000 to January 2026. The search strategy included combinations of the following keywords: “obesity”, “morbid obesity”, “cutaneous manifestations”, “acanthosis nigricans”, “skin tags”, “psoriasis”, “hidradenitis suppurativa”, “insulin resistance”, “adipokines”, and “transepidermal water loss”.

No formal language restrictions were applied. However, the review primarily included articles available in English. Both experimental and clinical studies were considered, with emphasis on studies involving human populations when assessing clinical relevance. Reference lists of relevant articles and key clinical guidelines were also screened to identify additional sources.

The literature selection process consisted of an initial screening of titles and abstracts, followed by full-text evaluation of potentially relevant articles. An initial pool of records was identified through database searches. However, due to the narrative design of the review, a formal PRISMA flow diagram was not constructed. After screening and eligibility assessment, 63 articles were included in the final analysis.

We included clinical studies, observational studies, randomized trials and relevant review articles. Priority was given to studies reporting clinically relevant outcomes and to those with larger or more representative populations when available. In cases of potential overlap between study populations, preference was given to the most comprehensive or most recent dataset. In cases of potential overlap between study populations, preference was given to the most comprehensive or most recent dataset.

Morbid obesity was defined as a body mass index (BMI) ≥40 kg/m^2^ or ≥35 kg/m^2^ in the presence of obesity-related comorbidities, in accordance with established clinical criteria. However, due to the limited availability of studies specifically focusing on morbid obesity, a substantial proportion of the included data was derived from general obesity populations, which was taken into account during the interpretation of the findings.

Studies conducted in both adult and pediatric populations were considered. Pediatric data are presented in a dedicated subsection to account for age-specific differences.

Exclusion criteria included publications that did not address the scope of the review or were not relevant to the metabolic–dermatologic interface.

Due to the narrative design of this review, a formal systematic review protocol was not applied. However, efforts were made to ensure a structured and transparent selection of the literature. No new human or animal data were collected. Therefore, ethical approval was not required.

**Figure 1:**
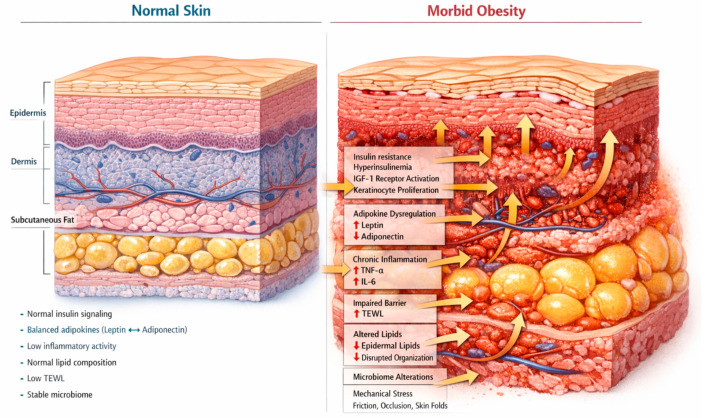
Structural and functional differences between normal skin and skin in morbid obesity. In morbid obesity, expansion of adipose tissue is associated with insulin resistance and hyperinsulinemia, leading to IGF-1 receptor activation and keratinocyte proliferation. Adipokine dysregulation (increased leptin, decreased adiponectin) and chronic low-grade inflammation (elevated TNF-α and IL-6) contribute to cutaneous changes. Impairment of epidermal barrier function is reflected by increased transepidermal water loss (TEWL) and altered lipid composition. Mechanical factors, including friction and occlusion within skin folds, further exacerbate barrier disruption and local inflammation.

**Figure 2:**
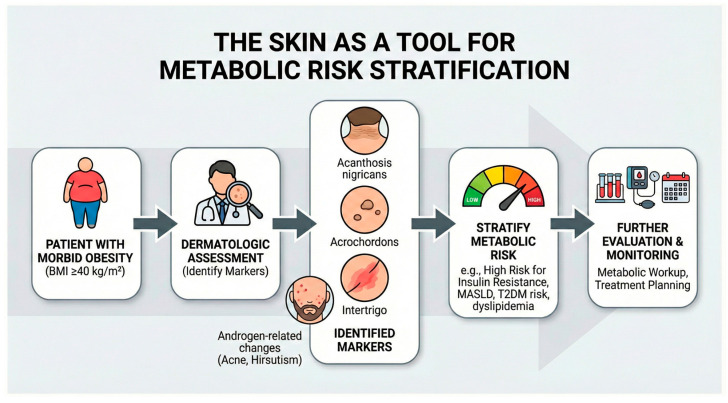
Conceptual framework illustrating the potential role of dermatologic assessment in metabolic risk stratification in morbid obesity. Selected dermatologic manifestations, including acanthosis nigricans, acrochordons, intertrigo, and androgen-related skin changes, may reflect underlying metabolic dysregulation associated with morbid obesity. Identification of these cutaneous markers during dermatologic examination may indicate the presence of insulin resistance and other metabolic abnormalities and may support further metabolic evaluation and clinical monitoring. Abbreviations: MASLD, metabolic dysfunction-associated steatotic liver disease; HbA1c, glycated hemoglobin.

**Figure 3:**
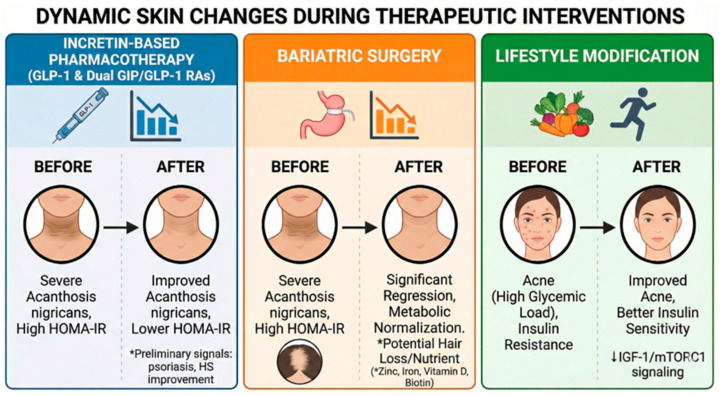
Schematic overview of changes in selected cutaneous manifestations during metabolic interventions in obesity. Improvement of metabolic parameters following pharmacological therapy with incretin-based agents, bariatric surgery, or lifestyle modification may be accompanied by changes in dermatologic manifestations associated with obesity. Regression of acanthosis nigricans has been reported following improvement in insulin resistance after metabolic treatment, while lifestyle-induced improvement in insulin sensitivity may contribute to a reduction in acne severity through modulation of insulin IGF-1-mTORC1 signaling pathways. Arrows indicate the direction of clinical change (before to after intervention). The asterisk (*) denotes preliminary or less well-established clinical observations, including potential improvement in psoriasis and hidradenitis suppurativa, as well as possible hair loss related to nutritional deficiencies.

Figure 1, Figure 2 and Figure 3 were generated using artificial intelligence-based tools. Figure 1 was created using ChatGPT (OpenAI), and Figure 2 and Figure 3 were generated using Gemini (Google; Nano Banana Pro), based on conceptual input provided by the authors. The authors reviewed and refined all generated images to ensure scientific accuracy and consistency with the content of the manuscript.

## 3. Results

### 3.1. Mechanisms

#### 3.1.1. Metabolic Mechanisms

Dermatologic manifestations observed in obesity do not represent a uniform group of “metabolic markers” but rather reflect distinct, partially overlapping pathophysiological axes. These include (1) insulin resistance-related changes driven by hyperinsulinemia and IGF signaling, (2) biomechanical and barrier-related alterations associated with increased skin fold depth and friction, and (3) systemic inflammatory dermatoses linked to immunometabolic dysregulation. Distinguishing these axes may improve the clinical interpretation of cutaneous findings and help align dermatologic observations with targeted diagnostic, preventive and risk stratification strategies.

In this framework, acanthosis nigricans and multiple acrochordons are primarily linked to the insulin resistance axis, intertriginous dermatoses reflect biomechanical and barrier-related stress, whereas inflammatory conditions such as psoriasis and hidradenitis suppurativa correspond to the systemic inflammatory axis.This section summarizes mechanistic pathways linking obesity to skin alterations. These processes provide biological plausibility but do not represent clinically validated biomarkers (Figure 1).

Insulin resistance plays a central role in cutaneous alterations associated with obesity, including acanthosis nigricans (AN). Elevated insulin levels can activate insulin-like growth factor-1 receptors (IGF-1R) on keratinocytes and dermal fibroblasts, leading to increased epidermal proliferation characteristic of this condition [14]. Clinical observations show that improving insulin sensitivity may result in partial regression of skin lesions in obese patients [15]. Observational data support the link between hyperinsulinemia and the development of acanthosis nigricans, reinforcing its biological basis in insulin resistance [16,17,18]. Insulin resistance is closely intertwined with adipokine dysregulation. Beyond its role in energy homeostasis, leptin exerts pro-inflammatory effects by activating immune cells and increasing the production of cytokines such as IL-6 and TNF-α [19]. In metabolic conditions characterized by insulin resistance, including obesity and polycystic ovary syndrome, increased adiposity has been associated with elevated circulating TNF-α and IL-6 levels, as well as alterations in leptin and adiponectin concentrations [20,21]. Adiponectin levels are reduced in obesity; adiponectin exerts anti-inflammatory and vasculoprotective effects, and its deficiency is associated with endothelial dysfunction and impaired vascular homeostasis [22].

Persistent low-grade inflammation characteristic of obesity modulates innate and adaptive immune pathways within peripheral tissues [5]. These systemic alterations create a pro-inflammatory milieu that may contribute to the modulation of cutaneous immune responses involved in inflammatory skin diseases.

Obesity is associated not only with systemic metabolic dysregulation but also with measurable alterations in epidermal barrier function. Population-based analyses demonstrate that increasing body mass index correlates with elevated transepidermal water loss (TEWL), indicating compromised barrier integrity [23]. Complementary molecular studies report reduced skin lipid content and decreased expression of lipogenic enzymes in individuals with obesity, which may impair stratum corneum lipid organization and barrier cohesion [24]. Given the central role of intercellular lipids in maintaining barrier structure, such alterations may predispose to increased permeability and local inflammatory activation. In addition, obesity-related mechanical factors, including increased skinfold depth and friction, may further modify local microenvironmental conditions and barrier performance [25].

Emerging evidence suggests that body mass index may be associated with alterations in cutaneous microbial community structure. Observational sequencing studies have reported differences in beta-diversity and relative abundance of selected bacterial taxa across BMI categories [26]. However, in inflammatory dermatoses strongly associated with obesity, such as hidradenitis suppurativa, microbial patterns appear to be driven predominantly by anatomical location and inflammatory status rather than BMI alone [27,28]. These findings indicate that obesity-related microenvironmental changes may interact with host–microbial dynamics without establishing a direct causal pathway.

Most available data derive from cross-sectional and observational studies. Randomized controlled trials evaluating changes in cutaneous barrier function or microbiome composition as predefined outcomes of obesity treatment are currently lacking, and dermatologic parameters have not been incorporated as primary endpoints in major metabolic trials [29].

Accordingly, these mechanisms should not be interpreted as direct evidence of clinical utility but rather as a biological framework that supports the classification of dermatologic findings into distinct pathophysiological axes.

#### 3.1.2. Hormonal Mechanisms

Obesity is associated with disturbances in multiple hormonal pathways that affect skin structure and function. Hyperinsulinemia, adipokine imbalance, altered androgen signaling and local glucocorticoid activation contribute to changes in epidermal proliferation, barrier integrity, and cutaneous immune responses.

Observational data support hyperinsulinemia as a key hormonal driver of obesity-related cutaneous changes [25], with experimental evidence indicating activation of proliferative signaling pathways in keratinocytes and dermal fibroblasts [14]. However, the available evidence is predominantly derived from observational and cross-sectional studies, and randomized controlled trials assessing dermatologic endpoints remain lacking.

Hyperinsulinemia may also contribute to functional hyperandrogenism. In insulin-resistant states, including obesity and polycystic ovary syndrome, hyperinsulinemia enhances ovarian androgen production and reduces sex hormone-binding globulin levels, increasing free androgen availability [30]. This may contribute to clinical phenotypes such as acne, seborrhea and hirsutism, reflecting systemic endocrine dysregulation rather than primary skin disease.

Adipokine imbalance constitutes an additional hormonal dimension of obesity-related skin alterations. Leptin levels correlate with adipose tissue mass and exert biological activity in the skin. Leptin receptors are expressed on keratinocytes and leptin modulates keratinocyte proliferation and cytokine production, supporting its pro-inflammatory role in the cutaneous microenvironment [31]. Broader analyses indicate that adipokines influence inflammatory pathways and epidermal homeostasis [9]. However, direct interventional evidence linking leptin modulation to improvement of cutaneous manifestations in morbid obesity remains limited.

Disturbances in glucocorticoid metabolism represent another component of hormonal dysregulation in obesity. Although systemic hypercortisolism is not typical, enhanced local glucocorticoid activation has been described. 11β-hydroxysteroid dehydrogenase type 1 (11β-HSD1) converts inactive cortisone into active cortisol, increasing intracellular glucocorticoid availability; increased 11β-HSD1 activity has been reported in obesity [32]. Imaging studies demonstrate thinning of the epidermis and dermis following glucocorticoid exposure, confirming their structural effects on the skin [33]. However, direct data on cutaneous 11β-HSD1 activity in morbid obesity remain limited. Collectively, hormonal dysregulation in obesity, encompassing insulin resistance, adipokine imbalance, androgen excess and altered glucocorticoid metabolism, may contribute to altered epidermal barrier function, impaired immune regulation, and increased susceptibility to infections and delayed wound healing [25]. These effects are multifactorial and cannot be attributed to a single pathway. Most available evidence derives from mechanistic and observational studies, while prospective trials with predefined dermatologic endpoints remain scarce.

#### 3.1.3. Biomechanical Mechanisms

In obesity, biomechanical factors within skin folds-including skin-to-skin friction, occlusion, and persistent moisture exposure-play a central role in the development of fold-related dermatoses. Clinical observations indicate that enlargement and deepening of skin folds in severe obesity are associated with an increased frequency and recurrent course of intertrigo [34].

Mechanical friction between opposing skin surfaces leads to repetitive microtrauma of the epidermis. Under conditions of sustained humidity and occlusion, the stratum corneum becomes macerated, resulting in reduced mechanical resilience and disruption of barrier integrity [35]. This compromised barrier facilitates penetration of irritants and microorganisms and promotes local inflammatory activation.

Occlusion within deep skin folds further increases local temperature and impairs ventilation, creating a microenvironment characterized by moisture retention and reduced aeration. Such conditions favor secondary bacterial and fungal colonization, contributing to chronicity and recurrence of inflammatory lesions [34,35]. These microenvironmental alterations may also interact with local microbial communities; however, available evidence suggests that microbial patterns are influenced predominantly by anatomical location and inflammatory status rather than body mass index alone [28].

In patients with morbid obesity, these biomechanical conditions may complicate local skin management in intertriginous areas and contribute to recurrent inflammation. Collectively, available data support a direct relationship between mechanical burden in severe obesity and structural barrier impairment, underscoring the clinical relevance of biomechanical factors in fold-associated dermatoses [34,35].

### 3.2. The Skin as a Tool for Risk Stratification

#### 3.2.1. Cutaneous Manifestations as Indicators of Metabolic Risk

Selected dermatologic manifestations observed in morbid obesity reflect systemic metabolic and inflammatory dysregulation and may reflect underlying metabolic and inflammatory dysregulation and have been proposed as potential clinically observable correlates of systemic disease burden. These findings are consistent with the interpretation of AN as a phenotypic correlate of metabolic dysfunction (Figure 2).

This section focuses on clinically observable dermatologic manifestations associated with obesity. These findings are discussed as potential clinical markers. However, their diagnostic and prognostic value has not been validated in prospective studies.

Acanthosis nigricans (AN) is strongly associated with insulin resistance and sustained hyperinsulinemia. Cross-sectional analyses demonstrate its association with increased prevalence and severity of metabolic dysfunction associated steatotic liver disease (MASLD)related hepatic steatosis [36]. These findings support the interpretation of AN as a phenotypic marker of metabolic dysfunction.

Multiple acrochordons have been associated with elevated HOMA-IR values, dyslipidemia, and abnormalities in liver enzymes [37]. Increased IGF-1 expression has been described in acrochordons [38]. Epidemiologic data further indicate a higher prevalence of skin tags among individuals with type 2 diabetes mellitus [39]. Collectively, these observations suggest that proliferative benign cutaneous lesions may reflect underlying metabolic dysregulation.

Severe psoriasis represents a chronic inflammatory dermatosis linked to increased cardiovascular risk. Population-based cohort analyses demonstrate that patients requiring systemic therapy exhibit a significantly elevated risk of myocardial infarction compared with individuals without psoriasis, independent of traditional cardiovascular risk factors [40]. This association indicates increased systemic disease burden.

Chronic and recurrent intertrigo reflects biomechanical barrier impairment associated with severe obesity [34,35]. Although not directly linked to cardiovascular endpoints, persistent fold dermatoses may indicate substantial mechanical and barrier-related burden and contribute to chronic inflammation within intertriginous areas.

These findings suggest that dermatologic assessment may provide additional context for the assessment of metabolic risk. However, most evidence remains observational, and prospective validation of independent predictive value is limited (Table 1).

#### 3.2.2. Potential Composite Indicators

Obesity is associated with measurable alterations in epidermal barrier integrity. Physiological studies demonstrate a positive correlation between BMI and transepidermal water loss (TEWL) [44]. Alterations in epidermal lipid composition have been reported in individuals with obesity [24]. In addition, facial physiological parameters and skin microbiome composition have been shown to vary according to BMI, including differences in sebum production, hydration, pH, and bacterial community structure [45]. These structural and microenvironmental alterations may contribute to local inflammatory activation and increased susceptibility to infection.

In contrast, epidermal barrier parameters, transepidermal water loss, lipid composition and microbiome alterations are measurable and provide insight into cutaneous responses to metabolic dysfunction; their clinical applicability remains limited. Standardized measurement protocols, validated reference ranges stratified by BMI, age, sex, and prospective data demonstrating responsiveness to therapeutic interventions are currently lacking.

Psoriasis is increasingly conceptualized as an immunometabolic disorder. Dysregulation of adipokine signaling and activation of inflammatory pathways contribute to systemic immune amplification in affected individuals [9,40]. This bidirectional interaction between metabolic and immune pathways provides a rationale for exploring psoriasis severity as a potential composite indicator of systemic inflammatory burden.

Clinical data evaluating GLP-1 receptor agonists in psoriasis include case reports, prospective cohorts, and small randomized controlled trials [46,47,48]. Although reductions in PASI have been reported in observational settings and selected interventional studies, large randomized trials validating cutaneous endpoints as predictors of cardiovascular outcomes are lacking.

Therefore, epidermal barrier parameters, transepidermal water loss, lipid composition, and microbiome alterations should currently be considered as emerging research directions rather than tools for routine clinical risk stratification or treatment monitoring, as their incremental value beyond established metabolic markers remains uncertain. 

### 3.3. Monitoring Obesity Treatment Through Cutaneous Assessment

Cutaneous manifestations may also provide clinically observable indicators of disease burden and treatment response during obesity therapy (Table 2, Figure 3).

#### 3.3.1. Pharmacological Treatment (GLP-1 Receptor Agonists, Tirzepatide)

GLP-1 receptor agonists improve metabolic parameters, including body weight and insulin resistance [29]. These systemic effects may be associated with secondary changes in obesity-related cutaneous manifestations.

Improvement of insulin resistance during GLP-1-based therapy has been associated with regression of acanthosis nigricans [41]. However, randomized trials of GLP-1-based therapies have not included regression of acanthosis nigricans as a predefined dermatologic endpoint.

Reduction in adipose tissue mass during GLP-1-based therapy is associated with changes in circulating adipokines [29]. These changes may indirectly influence cutaneous inflammatory activity. Nevertheless, obesity pharmacotherapy trials have not systematically evaluated dermatologic outcomes, and high-quality interventional data remain limited.

Clinical evidence evaluating GLP-1 receptor agonists (GLP-1RAs) in psoriasis includes case reports, prospective cohort studies, randomized controlled trials, and systematic reviews. Observational studies generally report reductions in PASI scores accompanied by decreased dermal T-cell infiltration and reduced IL-17 and IL-23 expression, whereas randomized data remain limited and partially heterogeneous [46,47,48]. These findings suggest that improvements in psoriasis observed during GLP-1-based therapy may be associated with metabolic improvement, particularly weight reduction and decreased systemic inflammation, rather than clearly established direct dermatologic effects of these agents.

Although severe psoriasis is associated with increased cardiovascular risk, psoriasis severity itself has not been validated as a surrogate cardiovascular endpoint.

An open-label randomized clinical trial evaluating semaglutide in obese patients with type 2 diabetes mellitus and psoriasis demonstrated significant improvement in psoriatic lesion severity alongside metabolic improvement [47]. However, the absence of blinding and the limited sample size restrict causal inference regarding direct antipsoriatic efficacy.

Beyond psoriasis, hidradenitis suppurativa (HS) represents another inflammatory dermatosis strongly associated with obesity and metabolic dysregulation. Despite the strong epidemiologic association between hidradenitis suppurativa and metabolic dysfunction [42,43], randomized controlled trials evaluating GLP-1 receptor agonists specifically in HS are currently lacking.

Available reports describing improvement of HS during GLP-1-based therapy are limited to case reports and small uncontrolled series, and may reflect secondary effects of weight loss and reduction in systemic inflammation rather than direct drug-specific action, precluding definitive therapeutic conclusions.

Taken together, current evidence suggests that metabolic improvement induced by GLP-1-based therapies may be associated with attenuation of selected obesity-associated dermatoses. However, adequately powered, blinded randomized trials with predefined dermatologic endpoints are required to determine whether these agents exert direct disease-modifying effects in psoriasis or hidradenitis suppurativa.

#### 3.3.2. Bariatric Surgery

Bariatric surgery leads to rapid improvement in insulin resistance, reflected by reductions in fasting insulin and HOMA-IR that may precede maximal weight loss. In a prospective cohort of obese patients undergoing laparoscopic sleeve gastrectomy (LSG), metabolic improvement at 3 months was accompanied by marked regression of obesity-associated acanthosis nigricans (OB_AN). Reduction in AN severity correlated with changes in BMI, insulin resistance indices, testosterone levels, and objective parameters of epidermal thickness and pigmentation assessed by reflectance confocal microscopy, indicating an association between metabolic normalization and structural cutaneous remodeling [49].

Mechanistically, attenuation of hyperinsulinemia may reduce insulin/IGF-1-mediated keratinocyte proliferation and melanogenesis, processes implicated in AN pathogenesis. Clinically, AN may therefore represent a dynamic and non-invasive correlate of early metabolic response after bariatric intervention; however, its validity as a standardized treatment endpoint has not been established in randomized controlled trials.

Independent of metabolic improvement, morbid obesity itself is associated with impaired surgical wound healing. Excess adipose tissue contributes to tissue hypoxia, increased mechanical tension, and a chronic pro-inflammatory milieu characterized by elevated TNF-α and IL-6, while microvascular dysfunction and insulin resistance impair fibroblast activity and keratinocyte proliferation. Clinically, severe obesity increases the risk of wound dehiscence, surgical site infection, and delayed epithelialization in the perioperative period [50].

In the early postoperative phase, structural cutaneous changes parallel metabolic improvement, including normalization of epidermal architecture and pigmentation [49]. These observations suggest that cutaneous remodeling after bariatric surgery reflects improved insulin signaling and modulation of local inflammatory and microcirculatory pathways.

In the long-term postoperative period, bariatric procedures, particularly those with a malabsorptive component, are associated with micronutrient deficiencies due to reduced intake and altered gastrointestinal absorption. Deficiencies in zinc, iron, vitamin D, and biotin affect hair follicle cycling and keratinocyte differentiation and are clinically associated with telogen effluvium, diffuse hair thinning, nail fragility, and xerosis following rapid weight loss [51,52]. Mechanistically, zinc and iron depletion impair matrix–cell proliferation and keratin synthesis. Clinically, structured biochemical monitoring and targeted supplementation are required to prevent dermatologic complications [51].

Importantly, no randomized controlled trials have used validated dermatologic scoring systems as primary endpoints in bariatric cohorts. Available evidence derives predominantly from observational studies [49]. Consequently, although cutaneous changes appear to parallel metabolic recovery, the role of dermatologic indices as formal tools for treatment monitoring requires confirmation in adequately powered interventional studies. These observations suggest that dermatologic changes following bariatric surgery may reflect both early metabolically driven responses (e.g., regression of acanthosis nigricans) and later treatment-associated effects related to micronutrient deficiencies.

#### 3.3.3. Lifestyle Modification

Lifestyle modification, particularly adherence to a low-glycemic-load diet, improves insulin sensitivity and reduces circulating insulin levels, which has been associated with significant clinical improvement in acne severity in randomized trials and meta-analytic analyses [53]. Hyperinsulinemia enhances insulin-like growth factor-1 (IGF-1) signaling and activates the nutrient-sensitive kinase mTORC1, promoting keratinocyte proliferation and sebaceous lipogenesis through downstream mediators such as SREBP-1, thereby contributing to acne pathogenesis [54]. Attenuation of the insulin-IGF-1-mTORC1 axis provides a biologically plausible explanation for the observed clinical benefit of dietary intervention in acne [53,54].

Obesity-related insulin resistance is accompanied by chronic low-grade inflammation and altered adipokine signaling. As previously discussed, adipokines such as leptin, adiponectin, TNF-α, and IL-6 modulate keratinocyte proliferation, differentiation, and inflammatory responses within the skin microenvironment, linking systemic metabolic imbalance with cutaneous homeostasis [9]. Improvement of insulin sensitivity through lifestyle intervention may therefore influence not only sebaceous activity but also broader inflammatory and barrier-related processes.

However, in contrast to acne—where validated severity indices have been used in interventional trials—lifestyle studies have not incorporated standardized dermatologic endpoints for other obesity-associated dermatoses. Consequently, while acne severity may represent a measurable cutaneous correlate of dietary metabolic modulation, evidence supporting the use of additional skin parameters as monitoring tools in lifestyle-based obesity treatment remains limited.

Dermatologic manifestations associated with obesity may differ in their temporal dynamics in response to therapeutic interventions. Certain features, such as acanthosis nigricans, may demonstrate relatively rapid improvement following metabolic normalization, particularly after bariatric surgery, reflecting early changes in insulin resistance. In contrast, other cutaneous alterations, including inflammatory dermatoses and barrier-related abnormalities, may exhibit slower or indirect responses, corresponding to more complex immunometabolic and structural remodeling. Additionally, some dermatologic findings observed during treatment, such as telogen effluvium or xerosis, may reflect treatment-related effects, including micronutrient deficiencies, rather than direct indicators of metabolic improvement.

### 3.4. Pediatric and Adolescent Phenotype

#### 3.4.1. Acanthosis Nigricans as a Marker of Insulin Resistance in Youth

Acanthosis nigricans (AN) in children and adolescents with overweight or obesity is associated with hyperinsulinemia and insulin resistance; the proposed mechanism involves insulin-mediated activation of IGF-1 signaling, promoting keratinocyte and dermal fibroblast proliferation and resulting in epidermal hyperplasia [55].

In a cross-sectional study of 139 overweight adolescents (BMI ≥ 85th percentile), the presence of AN was associated with significantly higher fasting insulin concentrations and HOMA-IR values compared with adolescents without AN (26.4 ± 16.3 vs. 19.0 ± 10.3 μIU/mL; 5.59 ± 3.51 vs. 4.00 ± 2.29; *p* = 0.003 for both). Insulin resistance (HOMA-IR ≥ 90th percentile) was more prevalent in adolescents with AN (62.7% vs. 38.9%; *p* = 0.008), and AN increased the odds of insulin resistance (OR 2.59; *p* = 0.009).

In obese children, HOMA-IR values were significantly higher in those with AN than in those without AN (5.74 ± 4.71 vs. 2.14 ± 0.86; *p* < 0.001). HOMA-IR increased across categories of AN severity, and receiver operating characteristic analysis demonstrated an area under the curve of 0.765 for AN in predicting insulin resistance. A severity score ≥ 3 was associated with a specificity of 83.9% [41].

Available pediatric data demonstrate a consistent cross-sectional association between AN and biochemical markers of insulin resistance in youth with excess adiposity [36,41]. Prospective studies evaluating its independent longitudinal prognostic value are lacking.

#### 3.4.2. Other Cutaneous Manifestations in Pediatric Obesity

Beyond acanthosis nigricans, several additional dermatologic conditions have been reported in children and adolescents with obesity, some of which demonstrate measurable metabolic associations.

In a cross-sectional study of 103 children and adolescents with obesity, striae distensae (63–71%), keratosis pilaris (63–65%), acne vulgaris (39–41%), acrochordons (25%), and acanthosis nigricans (45%) were the most prevalent findings. Importantly, HOMA-IR values were significantly associated not only with acanthosis nigricans (*p* = 0.047), but also with keratosis pilaris (*p* = 0.019) and acne vulgaris (*p* < 0.001) [56]. These findings suggest that selected cutaneous phenotypes in pediatric obesity correlate with objective biochemical markers of insulin resistance.

Psoriasis has also been linked to metabolic abnormalities in pre-pubertal children. In a prospective study of 60 children aged 3–10 years with psoriasis, 30% met criteria for metabolic syndrome, and 27% demonstrated insulin resistance defined as HOMA-IR ≥ 90th percentile. The prevalence of metabolic syndrome in this cohort was significantly higher than that reported in the general European pediatric population (30% vs. 4.8%; *p* < 0.001) [57]. These data support an association between pediatric psoriasis and systemic metabolic dysregulation early in life.

Hidradenitis suppurativa (HS), although less common in childhood than in adulthood, demonstrates a consistent epidemiologic association with obesity. In a scoping review including 2911 pediatric HS patients, 42.3% were classified as obese (BMI > 30 kg/m^2^) and 14% as overweight. Adjusted analyses from included studies reported that adolescents with HS were significantly more likely to be obese compared with controls (OR 2.48; 95% CI 2.38–2.59; *p* < 0.001) [58]. While causality cannot be inferred, these data demonstrate a robust association between pediatric HS and excess adiposity.

Acne vulgaris has similarly been linked to metabolic alterations in adolescents and young adults. Although pediatric obesity-specific mechanistic trials are limited, case-based and clinical data indicate the coexistence of acne with metabolic syndrome components and insulin resistance [59]. In the pediatric obesity cohort described above, acne prevalence correlated with HOMA-IR and fasting insulin levels [56]. These observations suggest that metabolic dysregulation may contribute to acne severity in youth with excess adiposity.

Collectively, available pediatric evidence indicates that selected inflammatory and proliferative skin conditions—particularly psoriasis, hidradenitis suppurativa, keratosis pilaris, and acne—occur with increased frequency in children with obesity and, in several cases, correlate with objective metabolic parameters [56,57,58,59]. However, most available data derive from cross-sectional or observational studies, and prospective longitudinal analyses evaluating independent predictive value for long-term metabolic outcomes remain lacking.

#### 3.4.3. Monitoring and Long-Term Outcomes

Cutaneous markers such as acanthosis nigricans (AN) reflect underlying hyperinsulinemia and insulin resistance in children and adolescents with excess adiposity [41]. Although prospective studies directly linking AN to long-term cardiometabolic outcomes are lacking, longitudinal evidence supports the dynamic and modifiable nature of insulin resistance in youth.

In the IDEFICS cohort, higher BMI z-score and waist circumference were strong prospective predictors of incident insulin resistance (HOMA-IR ≥ 95th percentile), and longitudinal reductions in BMI were accompanied by parallel declines in HOMA-IR values [60]. These findings indicate that insulin resistance in childhood tracks with weight trajectory and may improve with effective lifestyle modification.

Long-term registry data further demonstrate that effective pediatric obesity treatment—particularly achieving obesity remission or substantial BMI reduction—is associated with significantly lower risks of type 2 diabetes, dyslipidemia, hypertension, bariatric surgery, and premature mortality in young adulthood [61]. The magnitude of risk reduction appears proportional to treatment response.

Collectively, while AN should be interpreted primarily as a cross-sectional clinical marker of insulin resistance, its presence may identify children who warrant structured metabolic monitoring and early weight-management intervention. However, prospective studies assessing whether changes in AN severity independently predict long-term cardiometabolic outcomes are currently unavailable.

### 3.5. Quality of Available Evidence

The current body of evidence linking dermatologic manifestations with morbid obesity is characterized predominantly by observational study designs. Most associations between acanthosis nigricans and insulin resistance derive from cross-sectional analyses [16,17,41]. Associations between acanthosis nigricans and broader metabolic dysfunction, including metabolic dysfunction-associated steatotic liver disease (MASLD), are likewise largely based on observational data [36]. Similarly, studies linking BMI with epidermal barrier impairment, altered lipid composition, and microbiome variability are derived from population-based or sequencing studies of observational design [23,24,26]. Although these data consistently demonstrate correlations, they do not establish temporal sequence or causality.

Interventional evidence remains limited with respect to dermatologic endpoints. Randomized controlled trials of GLP-1 receptor agonists and dual incretin-based therapies have consistently demonstrated significant metabolic improvement; however, dermatologic outcomes were not incorporated as predefined endpoints in trial protocols [29]. Reported improvements in inflammatory or proliferative skin conditions derive primarily from secondary analyses, small prospective cohorts, or uncontrolled settings [46,49]. Bariatric surgery studies describing regression of acanthosis nigricans are prospective but observational in nature and lack randomized comparators [49]. To date, no adequately powered randomized controlled trial in obesity has used validated dermatologic scoring systems as primary endpoints.

A further limitation concerns the absence of standardized and externally validated dermatologic indices for metabolic risk stratification. While severity grading of acanthosis nigricans has been correlated with HOMA-IR values in pediatric cohorts [41], no composite dermatologic–metabolic index has been developed or externally validated. Likewise, parameters such as transepidermal water loss or epidermal lipid alterations are not integrated into established metabolic risk algorithms [23,24,45]. Current diabetes and obesity care frameworks rely exclusively on biochemical and clinical parameters rather than cutaneous markers [7].

Finally, available studies rarely differentiate class III obesity as a distinct metabolic phenotype. Few analyses stratify dermatologic outcomes according to the presence or absence of overt type 2 diabetes, MASLD, or other advanced metabolic complications within the BMI ≥ 40 kg/m^2^ subgroup. Consequently, it remains unclear whether dermatologic markers provide incremental prognostic value beyond established metabolic indicators in severe obesity.

Collectively, the existing evidence supports biologically plausible associations between cutaneous manifestations and metabolic dysfunction. However, the predominance of cross-sectional data, absence of randomized dermatologic endpoints, lack of validated scoring systems, and limited phenotypic stratification constrain definitive translational conclusions.

### 3.6. Research Gaps

Despite consistent associations between dermatologic manifestations and metabolic dysfunction, the available evidence remains methodologically limited.

First, most data linking acanthosis nigricans, acrochordons, and other obesity-associated dermatoses with insulin resistance derive predominantly from cross-sectional or observational studies [16,17,18,36,41]. Although correlations with HOMA-IR, metabolic syndrome, and metabolic dysfunction-associated steatotic liver disease (MASLD) have been demonstrated, these study designs do not establish temporal sequence or independent prognostic value. To date, no prospective cohort studies have evaluated whether longitudinal changes in lesion severity predict incident type 2 diabetes, MASLD progression, or cardiovascular outcomes.

Second, interventional trials in obesity have not incorporated predefined dermatologic endpoints. Randomized controlled trials of GLP-1 receptor agonists and dual incretin-based therapies have demonstrated significant metabolic improvement; however, structured assessment of acanthosis nigricans severity, epidermal barrier parameters (e.g., transepidermal water loss), or microbiome composition was not included in trial protocols [29]. Bariatric surgery studies reporting regression of acanthosis nigricans are prospective but observational and lack randomized comparators [49]. Accordingly, the role of cutaneous markers as validated tools for treatment monitoring remains unconfirmed.

Third, no standardized or externally validated composite dermatologic index quantifying metabolic burden has been developed. While severity grading of acanthosis nigricans correlates with insulin resistance indices in pediatric populations [41], dermatologic parameters are not incorporated into established metabolic risk algorithms or diabetes care frameworks [7]. The absence of validated scoring systems limits the integration of cutaneous findings into structured clinical decision-making pathways.

Fourth, phenotypic stratification within class III obesity (BMI ≥ 40 kg/m^2^) remains insufficient. Available studies typically evaluate associations with insulin resistance or metabolic syndrome [18,25,42] but do not provide detailed subgroup analyses according to the presence or absence of overt type 2 diabetes, MASLD, or other advanced metabolic complications. Consequently, the incremental prognostic value of dermatologic markers in severe obesity beyond established metabolic diagnoses has not been determined.

Finally, translational studies integrating dermal white adipose tissue biology, adipokine signaling, microvascular dysfunction, and clinical dermatologic outcomes in morbid obesity populations remain limited [9,10,11,25]. Mechanistic insights are frequently extrapolated from systemic metabolic data rather than derived from integrated cutaneous–metabolic analyses.

Addressing these limitations will be necessary before dermatologic assessment can be incorporated into structured metabolic risk stratification and treatment-monitoring frameworks.

## 4. Discussion

This review summarizes current evidence showing that the skin may reflect metabolic disturbances in obesity, including its severe forms. Adipose tissue is now seen as an active immunometabolic organ. Because of this, more attention is being given to other tissues, including the skin, as possible indicators of metabolic burden. Current data suggest that some skin changes are linked to key metabolic processes, especially insulin resistance, adipokine imbalance, and chronic low-grade inflammation.

Among the analyzed skin findings, acanthosis nigricans is the most consistently reported feature associated with hyperinsulinemia. Patients with this condition often have higher fasting insulin levels and higher HOMA-IR values, which indicate impaired insulin signaling. Multiple acrochordons have also been linked to metabolic abnormalities such as dyslipidemia, abnormal liver enzymes, and type 2 diabetes. This suggests that these benign skin lesions may reflect disturbances in insulin and growth factor pathways.

Higher body mass index is also associated with changes in the structure and function of the skin barrier. Studies show increased transepidermal water loss, changes in skin lipid composition, and alterations in the skin microbiome in people with obesity. This means that metabolic disturbances affect the local skin environment. In addition, mechanical factors, especially in skin folds, can promote inflammation and weaken the skin barrier. Overall, these findings show that the skin responds to metabolic, hormonal, inflammatory, and mechanical factors.

However, several limitations reduce the clinical applicability of these findings. Most studies are cross-sectional or observational, so they do not allow causal conclusions. Dermatologic outcomes are rarely included as endpoints in major metabolic trials. In addition, no validated composite indices combining dermatologic and metabolic parameters exist. It is also important to note that although this review focuses on morbid obesity, many studies include general obesity without separating severity. Therefore, the findings may not fully apply to patients with BMI ≥ 40 kg/m^2^ and should be interpreted with caution.

Moreover, it should be emphasized that the majority of available evidence is associative and does not allow for causal inference. Many dermatologic findings discussed in this review, including acanthosis nigricans, acrochordons, psoriasis and hidradenitis suppurativa, are closely linked to obesity and metabolic dysfunction. However, their independent predictive value beyond established clinical and biochemical markers (such as BMI, waist circumference, HbA1c, and lipid profile) remains uncertain.

Additionally, residual confounding is likely to influence these associations. Factors such as age, sex, ethnicity, comorbid conditions (including polycystic ovary syndrome), medication use and socioeconomic status may affect both the occurrence of dermatologic manifestations and metabolic outcomes.

Overall, skin changes in patients with obesity seem to reflect underlying metabolic and hormonal disturbances. They may serve as additional, non-invasive clinical clues. However, they should not be treated as independent tools for risk assessment or for monitoring treatment. At present, it is unclear whether they provide additional predictive value beyond standard metabolic markers.

To further enhance the clinical applicability of these observations, a prototype composite “obesity–dermatology index” may be proposed. Such an index could integrate selected dermatologic features representing different pathophysiological domains, including insulin resistance-associated markers (e.g., acanthosis nigricans severity), proliferative lesions (e.g., number of acrochordons), biomechanical/barrier-related findings (e.g., presence and severity of intertrigo) and inflammatory dermatoses (e.g., psoriasis or hidradenitis suppurativa severity).

Each component could be assessed using simple semi-quantitative clinical scales, allowing the generation of an aggregate score reflecting overall dermatologic burden associated with obesity.

Prospective validation of such an index would require correlation with established metabolic parameters, including HOMA-IR, HbA1c, lipid profile, and markers of hepatic steatosis, as well as evaluation of its predictive value for cardiometabolic outcomes over time.

Importantly, such an approach would need to account for confounding variables, including BMI, age, sex, and treatment status, and also demonstrate reproducibility and interobserver reliability.

Although currently conceptual, this framework may provide a basis for future studies aimed at integrating dermatologic assessment into metabolic risk stratification and treatment monitoring. Future studies should be prospective and well-designed. They should assess whether changes in skin findings correlate with metabolic outcomes and treatment response over time. Including standardized dermatologic scoring systems in clinical trials may help determine whether skin findings add meaningful information beyond routine biochemical markers.

Recent studies indicate that artificial intelligence systems for image recognition can achieve high diagnostic performance in dermatology and medical image analysis. Such systems have been applied to the classification and assessment of skin lesions based on clinical images [62,63]. Obesity-associated cutaneous manifestations, including acanthosis nigricans, acrochordons, intertrigo and inflammatory dermatoses, present with distinct visual features that can be captured on clinical images. In this context, artificial intelligence-based approaches may enable more standardized detection and objective assessment of lesion severity, with potential application in longitudinal evaluation. From a clinical perspective, this may support more consistent dermatologic assessment and facilitate further investigation into their potential role in metabolic risk stratification and treatment monitoring. However, clinical applicability in this specific context remains to be established and requires prospective validation.

From a clinical perspective, selected dermatologic findings may serve as adjunctive prompts for further evaluation rather than diagnostic criteria. For example, acanthosis nigricans and multiple acrochordons may support consideration of metabolic screening, whereas intertrigo may indicate the need for targeted fold-care strategies and evaluation of mechanical factors. Importantly, the strength of the evidence underlying these associations remains variable and largely relies on observational data.

## 5. Conclusions

Morbid obesity is characterized by chronic low-grade inflammation, adipokine dysregulation, and insulin resistance, processes that influence peripheral tissues, including the skin [5,9,25]. Evidence synthesized in this review indicates that cutaneous alterations reflect underlying metabolic and hormonal imbalance rather than representing isolated dermatologic phenomena.

Acanthosis nigricans remains the most consistently documented dermatologic correlate of hyperinsulinemia and insulin resistance. Cross-sectional studies demonstrate higher HOMA-IR values in affected individuals, and associations with metabolic syndrome and metabolic dysfunction-associated steatotic liver disease have been reported [16,17,36,41]. Similar metabolic associations have been described for multiple acrochordons, including links with impaired glucose metabolism and dyslipidemia [37,38,39].

Beyond proliferative lesions, obesity is associated with measurable impairment of epidermal barrier integrity and altered lipid composition [23,24,44]. Observational data also suggest BMI-associated variation in cutaneous microbiome characteristics [26,45]. However, these findings are derived predominantly from cross-sectional or observational designs and do not establish causality.

Interventional data indicate that metabolic improvement following GLP-1-based pharmacotherapy or bariatric surgery may be accompanied by regression of acanthosis nigricans [29,49]. Nevertheless, randomized controlled trials have not incorporated predefined dermatologic endpoints, and composite dermatologic indices for metabolic risk stratification remain undeveloped and unvalidated. Current metabolic care frameworks rely exclusively on biochemical and clinical parameters rather than cutaneous markers [7].

In pediatric populations, acanthosis nigricans demonstrates a reproducible association with insulin resistance indices [41]; however, prospective longitudinal data evaluating independent prognostic value remain lacking.

Collectively, available evidence supports the interpretation of selected dermatologic manifestations as phenotypic markers of systemic metabolic dysfunction in morbid obesity. However, the predominance of observational data, absence of standardized scoring systems, and lack of randomized dermatologic endpoints limit definitive integration of cutaneous assessment into formal metabolic care algorithms [7]. At present, these dermatological findings should be interpreted as associative markers rather than validated clinical tools for risk stratification or treatment monitoring.

## Figures and Tables

**Table 1:** Dermatologic Markers of Metabolic Dysfunction in Morbid Obesity, summarized in relation to underlying pathophysiologic mechanisms, associated metabolic parameters, type of evidence, and their clinical interpretation. The presented associations are predominantly derived from observational studies and should be interpreted with caution.

Cutaneous Marker	Dominant Pathophysiologic Axis	Associated Metabolic Parameter	Clinical Interpretation	What Next? (Clinical Implication)	Strength of Evidence	Evidence Type	Key References
Acanthosis nigricans	Chronic hyperinsulinemia activating IGF-1R signaling (Ras/MAPK, PI3K/Akt)	Insulin resistance; elevated HOMA-IR; MASLD	Phenotypic correlate of insulin resistance; severity is associated with metabolic burden	Consider metabolic evaluation (insulin resistance, glucose	Moderate	Cross-sectional; prospective observational	[16,17,18,36,41]
Multiple acrochordons	Hyperinsulinemia; enhanced IGF-1 signaling	Insulin resistance; dyslipidemia; abnormal liver enzymes; T2DM	Associated with metabolic dysfunction	Consider metabolic screening (metabolic syndrome, dyslipidemia)	Low–moderate	Case–control; observational	[37,38,39]
Moderate to severe psoriasis	Systemic inflammatory activation;	Increased cardiometabolic risk;	associated with increased cardiometabolic risk	Consider cardiometabolic risk assessment according to guidelines	Moderate	Population-based cohort	[40]
Hidradenitis suppurativa	Chronic inflammatory dermatosis linked with adiposity and metabolic dysregulation	Metabolic syndrome; increased BMI	a reported association between adiposity and disease severity	Consider evaluation of metabolic comorbidities	Low–moderate	Meta-analysis; prospective cohort	[42,43]

**Table 2:** Cutaneous Manifestations as Indicators of Disease Burden or Treatment Response.

Manifestation	Clinical Context	Observed Dynamic Change	Intervention Type	Clinical Relevance	Evidence Type	Key References
Acanthosis nigricans regression	Morbid obesity with insulin resistance	Reduction in lesion severity paralleling improved HOMA-IR and BMI	GLP-1-based therapy; bariatric surgery (LSG)	Potential non-invasive marker of early metabolic response	Prospective cohort; secondary analyses	[29,49]
Psoriasis severity (PASI reduction)	Obesity with inflammatory dermatosis	Variable reduction in PASI during metabolic therapy	GLP-1 receptor agonists	Possible metabolic inflammatory coupling; not validated as CV surrogate	Case reports; small RCTs; scoping review	[46,47,48]
Recurrent intertrigo	Severe central/mechanical obesity	Persistence correlates with fold depth and mechanical burden	Weight reduction	Marker of biomechanical and barrier stress	Clinical review; observational	[34,35]
Impaired wound healing	Severe obesity; perioperative setting	Increased dehiscence and infection risk	Surgical context	Reflects systemic inflammatory and metabolic dysfunction	Review data	[50]
Post-bariatric telogen effluvium	Rapid postoperative weight loss	Diffuse hair thinning linked to micronutrient deficiency	Bariatric surgery	Requires biochemical monitoring and supplementation	Observational	[51,52]

## Data Availability

No new data were created or analyzed in this study. Data sharing is not applicable to this article.

## Abbreviations

The following abbreviations are used in this manuscript:

AN	Acanthosis nigricans
BMI	Body mass index
CI	Confidence interval
CKD	Chronic kidney disease
DLQI	Dermatology Life Quality Index
GIP	Glucose-dependent insulinotropic polypeptide
GLP-1	Glucagon-like peptide-1
GLP-1 RA	Glucagon-like peptide-1 receptor agonist
HbA1c	Glycated hemoglobin
HOMA-IR	Homeostatic Model Assessment of Insulin Resistance
HS	Hidradenitis suppurativa
IGF-1	Insulin-like growth factor-1
IGF-1R	Insulin-like growth factor-1 receptor
IL-6	Interleukin-6
LSG	Laparoscopic sleeve gastrectomy
MASLD	Metabolic dysfunction-associated steatotic liver disease
mTORC1	Mechanistic target of rapamycin complex 1
OCT	Optical coherence tomography
OR	Odds ratio
PASI	Psoriasis Area and Severity Index
PCOS	Polycystic ovary syndrome
PI3K	Phosphoinositide 3-kinase
RCT	Randomized controlled trial
Ras/MAPK	Rat sarcoma/mitogen-activated protein kinase pathway
SHBG	Sex hormone-binding globulin
TEWL	Transepidermal water loss
TNF-α	Tumor necrosis factor-alpha
11β-HSD1	11β-hydroxysteroid dehydrogenase type 1

## References

[1] World Health Organization (2020). Obesity and Overweight.

[2] Purnell J.Q., Feingold K.R., Anawalt B., Boyce A., Chrousos G., de Herder W.W., Dhatariya K., Dungan K., Hershman J.M., Hofland J., Kalra S. (2000). Definitions, classification, and epidemiology of obesity. Endotext.

[3] McGowan B., Ciudin A., Baker J.L., Busetto L., Dicker D., Frühbeck G., Goossens G.H., Monami M., Sbraccia P., Martinez-Tellez B. (2025). Framework for the pharmacological treatment of obesity and its complications from the European Association for the Study of Obesity (EASO). Nat. Med..

[4] Garvey W.T., Mechanick J.I., Brett E.M., Garber A.J., Hurley D.L., Jastreboff A.M., Nadolsky K., Pessah-Pollack R., Plodkowski R. (2016). American Association of Clinical Endocrinologists and American College of Endocrinology comprehensive clinical practice guidelines for medical care of patients with obesity. Endocr. Pract..

[5] Hotamisligil G.S. (2006). Inflammation and metabolic disorders. Nature.

[6] Dixon J.B. (2010). The effect of obesity on health outcomes. Mol. Cell. Endocrinol..

[7] American Diabetes Association Professional Practice Committee (2025). 2. Diagnosis and Classification of Diabetes: Standards of Care in Diabetes 2025. Diabetes Care.

[8] Slominski A.T., Zmijewski M.A., Plonka P.M., Szaflarski J.P., Paus R. (2018). How UV Light Touches the Brain and Endocrine System Through Skin, and Why. Endocrinology.

[9] Kovacs D., Fazekas F., Olah A., Töröcsik D. (2020). Adipokines in the skin and in dermatological diseases. Int. J. Mol. Sci..

[10] Kruglikov I.L., Scherer P.E. (2016). Skin aging: Are adipocytes the next target?. Aging.

[11] Alexander C.M., Kasza I., Yen C.L., Reeder S.B., Hernando D., Gallo R.L., Jahoda C.A.B., Horsley V., MacDougald O.A. (2015). Dermal white adipose tissue: A new component of the thermogenic response. J. Lipid Res..

[12] Nguyen A.V., Soulika A.M. (2019). The dynamics of the skin’s immune system. Int. J. Mol. Sci..

[13] Grice E.A., Segre J.A. (2011). The skin microbiome. Nat. Rev. Microbiol..

[14] Eggiman E., Feldman S.R. (2024). The underlying pathogenesis of obesity-associated acanthosis nigricans: A literature review. Discov. Med..

[15] Sun H., Wang X., Chen J., Gusdon A.M., Song K., Li L., Qu S. (2018). Melatonin Treatment Improves Insulin Resistance and Pigmentation in Obese Patients with Acanthosis Nigricans. Int. J. Endocrinol..

[16] Singh S.K., Agrawal N.K., Vishwakarma A.K. (2020). Association of Acanthosis Nigricans and Acrochordon with Insulin Resistance: A Cross-Sectional Hospital-Based Study. Indian J. Dermatol..

[17] Choudhary S., Srivastava A., Saoji V., Singh A., Verma I., Dhande S. (2023). Association of Acanthosis Nigricans with Metabolic Syndrome An Analytic Cross-Sectional Study. An. Bras. Dermatol..

[18] Karadağ A.S., You Y., Danarti R., Al-Khuzaei S., Chen W. (2018). Acanthosis nigricans and the metabolic syndrome. Clin. Dermatol..

[19] Fernández-Riejos P., Najib S., Santos-Alvarez J., Martín-Romero C., Pérez-Pérez A., González-Yanes C., Sánchez-Margalet V. (2010). Role of leptin in the activation of immune cells. Mediat. Inflamm..

[20] Fernández-Vallejo B., Jiménez Monteagudo F., Romero L., López Aznárez M.I., Romero Cobas M.C., Pérez-Martínez L. (2025). Cross-sectional analysis of IL-6, TNF-α, adiponectin, leptin, and klotho serum levels in relation to BMI among overweight and obese children aged 10–14 in La Rioja, Spain. Children.

[21] Cardoso N.S., Ribeiro V.B., Dutra S.G.V., Ferriani R.A., Gastaldi A.C., de Araújo J.E., de Souza H.C.D. (2020). Polycystic ovary syndrome associated with increased adiposity interferes with serum levels of TNF-α, IL-6 differently from leptin and adiponectin. Arch. Endocrinol. Metab..

[22] Zhao L., Fu Z., Liu Z. (2021). Adiponectin and cardiovascular disorders. Circ. Res..

[23] Yew Y.W., Mina T., Ng H.K., Lam B.C.C., Riboli E., Lee E.S., Lee J., Ngeow J., Elliott P., Thng S.T.G. (2023). Investigating Causal Relationships between Obesity and Skin Barrier Function in a Multi-Ethnic Asian Cohort. Int. J. Obes..

[24] Horie Y., Makihara H., Horikawa K., Takeshige F., Ibuki A., Satake T., Yasumura K., Maegawa J., Mitsui H., Ohashi K. (2018). Reduced Skin Lipid Content in Obese Japanese Women Mediated by Decreased Expression of Lipogenic Enzymes. PLoS ONE.

[25] Darlenski R., Mihaylova V., Handjieva-Darlenska T. (2022). The link between obesity and the skin. Front. Nutr..

[26] Brandwein M., Katz I., Katz A., Kohen R. (2019). Beyond the gut: Skin microbiome compositional changes are associated with BMI. Hum. Microbiome J..

[27] Ring H.C., Thorsen J., Saunte D.M.L., Lilje B., Bay L., Riis P.T., Larsen N., Andersen L.O., Nielsen H.V., Miller I.M. (2017). The follicular skin microbiome in patients with hidradenitis suppurativa and healthy controls. JAMA Dermatol..

[28] Wolk K., Join-Lambert O., Sabat R. (2020). Aetiology and pathogenesis of hidradenitis suppurativa. Br. J. Dermatol..

[29] Corrao S., Pollicino C., Maggio D., Torres A., Argano C. (2024). Tirzepatide against obesity and insulin resistance: Pathophysiological aspects and clinical evidence. Front. Endocrinol..

[30] Ibáñez L., Oberfield S.E., Witchel S., Auchus R.J., Chang R.J., Codner E., Dabadghao P., Darendeliler F., Elbarbary N.S., Gambineri A. (2017). An International Consortium Update: Pathophysiology, Diagnosis, and Treatment of Polycystic Ovarian Syndrome in Adolescence. Horm. Res. Paediatr..

[31] Poeggeler B., Schulz C., Pappolla M.A., Bodo E. (2009). Leptin and the skin: A new frontier. Exp. Dermatol..

[32] Wake D.J., Walker B.R. (2004). 11β-Hydroxysteroid dehydrogenase type 1 in obesity and the metabolic syndrome. Mol. Cell. Endocrinol..

[33] Aschoff R., Lang A., Koch E. (2022). Effects of intermittent treatment with topical corticosteroids and calcineurin inhibitors on epidermal and dermal thickness using optical coherence tomography and ultrasound. Skin Pharmacol. Physiol..

[34] Pera F., Suman C., Cosma M., Mazza S., Brunani A., Cancello R. (2025). Intertrigo in Severe Obesity: Clinical Insights and Outcomes with a New Antimicrobial Silver-Infused Breathable Fabric. J. Cosmet. Dermatol..

[35] Romanelli M., Voegeli D., Colboc H., Bassetto F., Janowska A., Scarpa C., Meaume S. (2023). The diagnosis, management and prevention of intertrigo in adults: A review. J. Wound Care.

[36] Sánchez-García A., Penados-Ovalle M.E., Rodríguez-Gutiérrez R., Colmeneros F.D.-G., González-González J.G. (2025). Acanthosis nigricans as a clinical risk marker for metabolic dysfunction-associated steatotic liver disease. Clin. Med. Insights Endocrinol. Diabetes.

[37] Gönülal M., Teker K. (2020). The role of impaired glucose and lipid metabolism and liver enzyme abnormalities in the formation of multiple acrochordons: A case-control study. Mucosa.

[38] Farag A.G.A., Abdu Allah A.M.K., El-Rebey H.S., Mohamed Ibraheem K.I., Mohamed A.S., Labeeb A.Z. (2019). Role of insulin-like growth factor-1 in skin tags: A clinical, genetic and immunohistochemical study in a sample of Egyptian patients. Clin. Cosmet. Investig. Dermatol..

[39] Saxena K., Zaidi A., Shaafie H.I., Singh N., Singh K.K. (2020). Skin tags in type 2 diabetes mellitus: A valuable marker. Int. J. Med. Biomed. Stud..

[40] Gelfand J.M., Neimann A.L., Shin D.B. (2006). Risk of myocardial infarction in patients with psoriasis. JAMA.

[41] Koh Y.K., Lee J.H., Kim E.Y., Moon K.R. (2016). Acanthosis Nigricans as a Clinical Predictor of Insulin Resistance in Obese Children. Pediatr. Gastroenterol. Hepatol. Nutr..

[42] Rodríguez-Zuñiga M.J.M., García-Perdomo H.A., Ortega-Loayza A.G. (2019). Association Between Hidradenitis Suppurativa and Metabolic Syndrome: A Systematic Review and Meta-analysis. Actas Dermo-Sifiliogr..

[43] Kromann C.B., Ibler K.S., Kristiansen V.B., Jemec G.B.E. (2014). The Influence of Body Weight on the Prevalence and Severity of Hidradenitis Suppurativa. Acta Derm. Venereol..

[44] Yudhistira M.Y., Kusumawardani A., Widhiati S., Mulianto N. (2022). The relationship between increased body mass index with transepidermal water loss and skin hydration. Clin. Cosmet. Investig. Dermatol..

[45] Ma L., Zhang H., Jia Q., Bai T., Yang S., Wang M., Li Y., Shao L. (2024). Facial Physiological Characteristics and Skin Microbiome Changes Are Associated with Body Mass Index (BMI). Clin. Cosmet. Investig. Dermatol..

[46] Karacabeyli D., Lacaille D. (2024). Glucagon-Like Peptide 1 Receptor Agonists in Patients with Inflammatory Arthritis or Psoriasis: A Scoping Review. J. Clin. Rheumatol..

[47] Petković-Dabić J., Binić I., Carić B., Božić L., Umičević-Šipka S., Bednarčuk N., Dabić S., Šitum M., Popović-Pejičić S., Stojiljković M.P. (2025). Effects of Semaglutide Treatment on Psoriatic Lesions in Obese Patients with Type 2 Diabetes Mellitus: An Open-Label, Randomized Clinical Trial. Biomolecules.

[48] Paschou I.A., Sali E., Paschou S.A., Psaltopoulou T., Nicolaidou E., Stratigos A.J. (2025). The effects of GLP-1 receptor agonists on inflammatory skin diseases: A comprehensive review. J. Eur. Acad. Dermatol. Venereol..

[49] Fu Z., Zeng J., Zhu L., Wang G., Li P., Li W., Song Z., Su Z.B., Sun X., Tang H. (2023). Clinical factors associated with remission of obesity-associated acanthosis nigricans after laparoscopic sleeve gastrectomy: A prospective cohort study. Int. J. Surg..

[50] Pierpont Y.N., Dinh T.P., Salas R.E., Johnson E.L., Wright T.G., Robson M.C., Payne W.G. (2014). Obesity and surgical wound healing: A current review. ISRN Obes..

[51] Smolarczyk K., Meczekalski B., Rudnicka E., Suchta K., Szeliga A. (2024). Association of Obesity and Bariatric Surgery on Hair Health. Medicina.

[52] Itthipanichpong Y., Damkerngsuntorn W., Tangkijngamvong N., Udomsawaengsup S., Boonchayaanant P., Kumtornrut C., Kerr S.J., Asawanonda P., Rerknimitr P. (2020). Skin manifestations after bariatric surgery. BMC Dermatol..

[53] Sakhaei R., Mohsenpour M.A. (2020). Low Glycemic Load or Index Diet in Association with Acne Vulgaris: A Systematic Review and Meta-Analysis. Clin. Cosmet. Investig. Dermatol..

[54] Melnik B.C. (2012). Diet in Acne: Further Evidence for the Role of Nutrient Signalling in Acne Pathogenesis. Acta Derm.-Venereol..

[55] Pollock S., Swamy M.R., Tremblay E.S., Shen L. (2022). Acanthosis nigricans in the pediatric population: A narrative review of the current approach to management in primary care. Pediatr. Med..

[56] Hasse L., Jamiolkowski D., Reschke F., Kapitzke K., Weiskorn J., Kordonouri O., Biester T., Ott H. (2023). Pediatric Obesity and Skin Disease: Cutaneous Findings and Associated Quality-of-Life Impairments in 103 Children and Adolescents with Obesity. Endocr. Connect..

[57] Caroppo F., Galderisi A., Ventura L., Belloni Fortina A. (2021). Metabolic Syndrome and Insulin Resistance in Pre-Pubertal Children with Psoriasis. Eur. J. Pediatr..

[58] Mehta S., Metko D., Lam J.M. (2025). Obesity in Pediatric Hidradenitis Suppurativa: A Scoping Review. Pediatr. Dermatol..

[59] Endres L.M., Bungau A.F., Tit D.M., Bungau G.S., Radu A., Diaconu C.C., Marin R.C. (2025). Acne Vulgaris Associated with Metabolic Syndrome: A Three-Case Series Highlighting Pathophysiological Links and Therapeutic Challenges. Diagnostics.

[60] Peplies J., Börnhorst C., Günther K., Fraterman A., Russo P., Veidebaum T., Tornaritis M., De Henauw S., Marild S., Molnar D. (2016). Longitudinal associations of lifestyle factors and weight status with insulin resistance (HOMA-IR) in preadolescent children: The large prospective cohort study IDEFICS. Int. J. Behav. Nutr. Phys. Act..

[61] Putri R.R., Danielsson P., Ekström N., Ericsson Å., Lindberg L., Marcus C., Hagman E. (2025). Effect of Pediatric Obesity Treatment on Long-Term Health. JAMA Pediatr..

[62] Boostani M., Bánvölgyi A., Zouboulis C.C., Goldfarb N., Suppa M., Goldust M., Lőrincz K., Kiss T., Nádudvari N., Holló P. (2025). Large language models in evaluating hidradenitis suppurativa from clinical images. J. Eur. Acad. Dermatol. Venereol..

[63] Boostani M., Bánvölgyi A., Goldust M., Cantisani C., Pietkiewicz P., Lőrincz K., Holló P., Wikonkál N.M., Paragh G., Kiss N. (2025). Diagnostic Performance of GPT-4o and Gemini Flash 2.0 in Acne and Rosacea. Int. J. Dermatol..

